# Binding abilities of polyaminocyclodextrins: polarimetric investigations and biological assays

**DOI:** 10.3762/bjoc.13.271

**Published:** 2017-12-18

**Authors:** Marco Russo, Daniele La Corte, Annalisa Pisciotta, Serena Riela, Rosa Alduina, Paolo Lo Meo

**Affiliations:** 1 Dipartimento di Scienze e Tecnologie Biologiche, Chimiche e Farmaceutiche (STEBICEF), University of Palermo, V.le delle Scienze ed. 17, 90128 Palermo, Italy; 2 ATeNCenter, University of Palermo, V.le delle Scienze ed. 18, 90128 Palermo, Italy

**Keywords:** aminocyclodextrins, binding properties, nitroanilines, pDNA, polarimetry, supramolecular chemistry

## Abstract

Three polyaminocyclodextrin materials, obtained by direct reaction between heptakis(6-deoxy-6-iodo)-β-cyclodextrin and the proper linear polyamines, were investigated for their binding properties, in order to assess their potential applications in biological systems, such as vectors for simultaneous drug and gene cellular uptake or alternatively for the protection of macromolecules. In particular, we exploited polarimetry to test their interaction with some model *p*-nitroaniline derivatives, chosen as probe guests. The data obtained indicate that binding inside the host cavity is mainly affected by interplay between Coulomb interactions and conformational restraints. Moreover, simultaneous interaction of the cationic polyamine pendant bush at the primary rim was positively assessed. Insights on quantitative aspects of the interaction between our materials and polyanions were investigated by studying the binding with sodium alginate. Finally, the complexation abilities of the same materials towards polynucleotides were assessed by studying their interaction with the model plasmid pUC19. Our results positively highlight the ability of our materials to exploit both the cavity and the polycationic branches, thus functioning as bimodal ligands.

## Introduction

Polyamine macromolecules have attracted a widespread interest for their potential applications in various fields. Linear or branched polyethyleneimine (PEI) polymers [1–5], as well as polypropyleneimine (PPI) [6–8] and polyamidoamine (PAMAM) [7–16] dendrimers, have been used as proton sponges, capping agents for the synthesis of noble metal nanoparticles, and systems for the complexation and cell transfection of genetic material [17–22]. In particular, the complexation and transfection of polynucleotides also have been successfully accomplished by means of polycationic cyclodextrin or calixarene derivatives, obtained by anchoring suitable polyammonium or imidazolium pendant groups onto the main macrocycle scaffold [23–28]. The latter example is interesting, because of the well-known ability of these macrocycles to form inclusion complexes with diverse organic guest molecules [29–35]. Cyclodextrins (CDs), in particular, constitute appealing systems due to their biocompatibility, which allowed them to be approved by the FDA as human friendly products [36]. Thus, polycationic CDs might be used in principle as bimodal ligands for the simultaneous internalization of a polynucleotide (interacting with the polycationic branches) and a further bioactive/drug molecule (included into the host cavity). The critical examination of the available literature suggests that one main drawback of this approach is the fact that the syntheses of the tailored macrocyclic ligands (as pure chemical species) reported so far are lengthy and expensive, affording low overall yields. Thus, a cheaper, more straightforward alternative route would be highly desirable.

Recently, we have prepared useful polyaminocyclodextrin materials (AmCDs) in high overall yields (> 90%) by simply reacting a heptakis(6-deoxy-6-halo)-β-CD with an excess of a suitable polyamine [37]. The reaction leads to the exhaustive nucleophilic displacement of the halogen atoms on the starting material [38–39]. However, the products obtained constitute complex mixtures of various inseparable derivatives, having a different number of polyamine branches linked to the CD scaffold, that are isolated as partial hydrohalides. In fact, the same polyamine unit can undergo multiple substitution reactions (a possible mechanistic scheme is depicted in Supporting Information File 1, Figure S1), on the same N atom or on different N atoms. The characterization of the materials by means of combined ESIMS, NMR and potentiometric titration techniques, enabled to determine the average number of pendant arms (<*n*_p_>) and hydrohalic acid molecules (<*n*_HX_>) per AmCD unit. The acid–base behaviour of these materials can be modelled as a mixture of independent virtual weak bases. We already have employed these products as capping agents for the preparation of silver nanocomposites [37], which in turn have been tested as catalysts for nitroarene reduction and as antimicrobial agents in synergism with classical antibiotics [40–41]. Moreover, the same products have been used for the synthesis of pH-responsive nanosponges [42].

**Figure 1:**
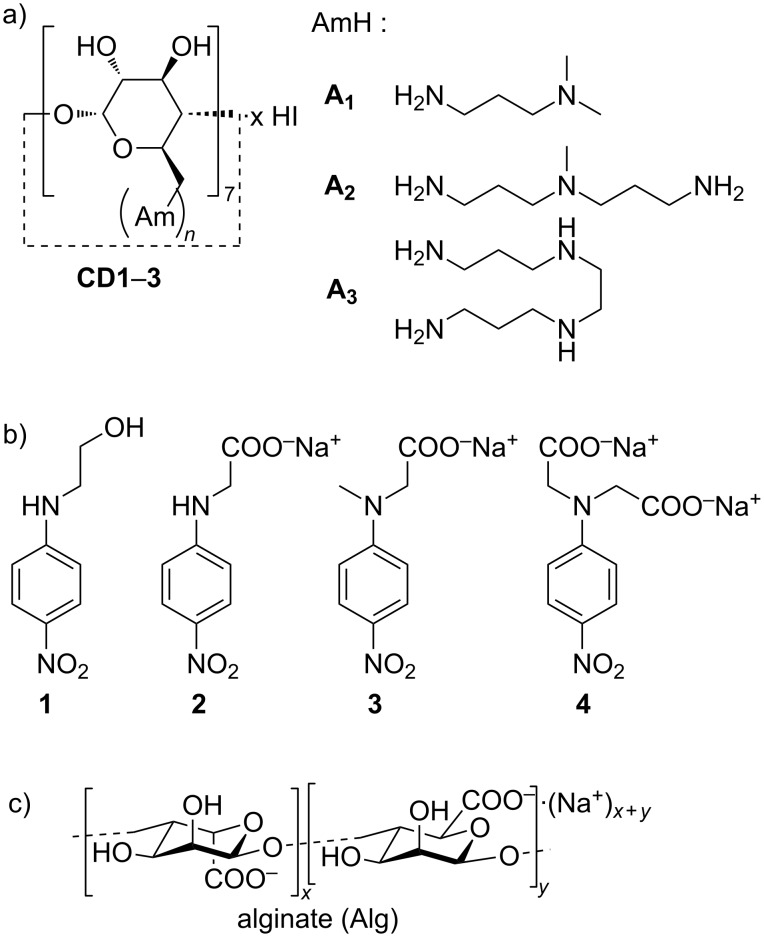
Structures of: a) AmCDs **CD1**–**3**; b) *p*-nitroaniline guests **1**–**4**; c) sodium alginate (Alg).

In view of possible further applications, the present work is aimed at verifying the abilities of AmCD materials synthesized by us to act as bimodal supramolecular ligands. To the best of our knowledge, the possible inclusion of a generic guest/drug molecule into CD derivatives bearing amine groups have been studied only occasionally [41,43–48]. Moreover, the interaction of polycationic CDs with polynucleotides has been mainly considered by targeting their abilities in gene internalization. However, a detailed examination of the relevant stoichiometric or thermodynamic aspects is lacking. We were interested in verifying how the presence of the dendrimer-like “bush” of polyamine pendants at the primary rim, and its protonation status as a function of the pH, might affect the inclusion properties of the main CD scaffold. At the same time, we wanted to clarify the microscopic features and quantitative aspects of the interaction between AmCDs and polyanions such as polynucleotides. For these purposes, we investigated by means of polarimetry the behaviour of materials **CD1**–**CD3** (Figure 1a), obtained in a previous work [37], with a set of selected neutral and anionic model *p*-nitroaniline derivatives **1**–**4** (Figure 1b) at different pH values. The materials chosen differ for the length and number of N atoms of the polyamine chains, and for the different average number of pendants per CD unit (i.e., 5.7, 6.1 and 4.5 for **CD1**, **CD2** and **CD3**, respectively). Moreover, we tested the potential ability of these materials to interact with polyanions by studying their behaviour towards sodium alginate (Alg, Figure 1c) chosen as suitable model compound. Finally, we performed some preliminary tests in order to assess their interaction with a model plasmid DNA (pUC19) and to evaluate whether they may influence the internalization of exogenous DNA in bacterial systems, in particular the Gram-negative model microorganism *Escherichia coli*.

## Results and Discussion

### Polarimetric behavior of AmCDs

**Figure 2:**
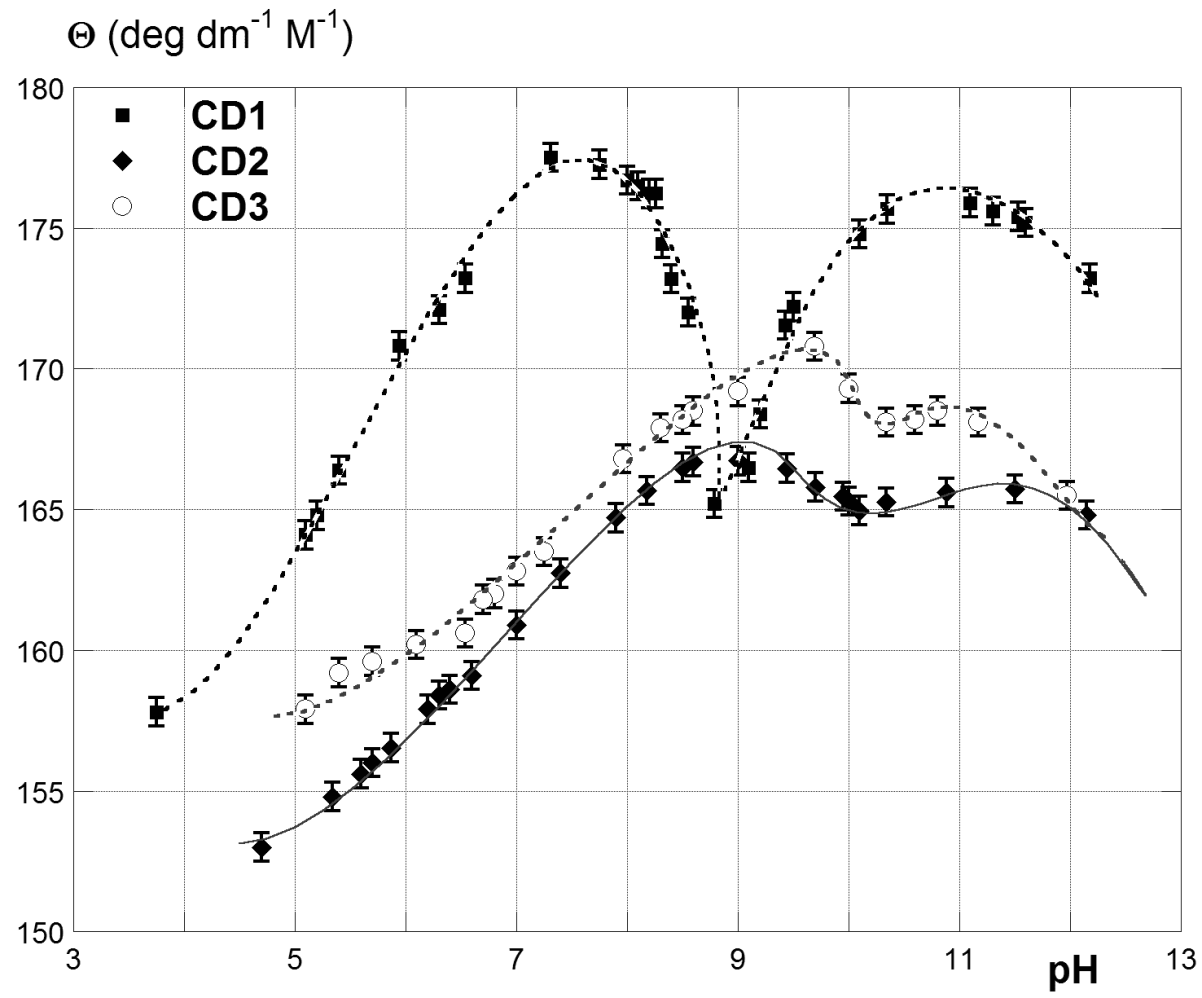
Trends of the molar optical rotation Θ of AmCDs **CD1**–**3** vs pH.

A preliminary investigation of the polarimetric behaviour of free AmCDs was a prerequisite before addressing their complexation abilities by means of polarimetry. Thus, the relevant molar optical rotations Θ were measured at different pH values, in order to study the possible effect of the progressive protonation of the polyamine groups and the results are depicted in Figure 2 (the complete data set is collected in the Supporting Information File 1, Table S2). Noticeably, Θ values were determined in the absence of any buffering or supporting electrolyte, simply by adjusting the pH with small amounts of added conc. HCl or NaOH. As we can easily notice, on varying the pH value of the solvent medium, and consequently the charge status of the AmCDs, the Θ values show a peculiar M-shaped trend. Starting from ca. pH 12, at which the products are almost uncharged, Θ initially increases, then decreases, then again rises up to a maximum value, and finally undergoes a regular decrease up to pH 5 and beyond. This behaviour is more pronounced for **CD1** than for the other two products.

**Figure 3:**
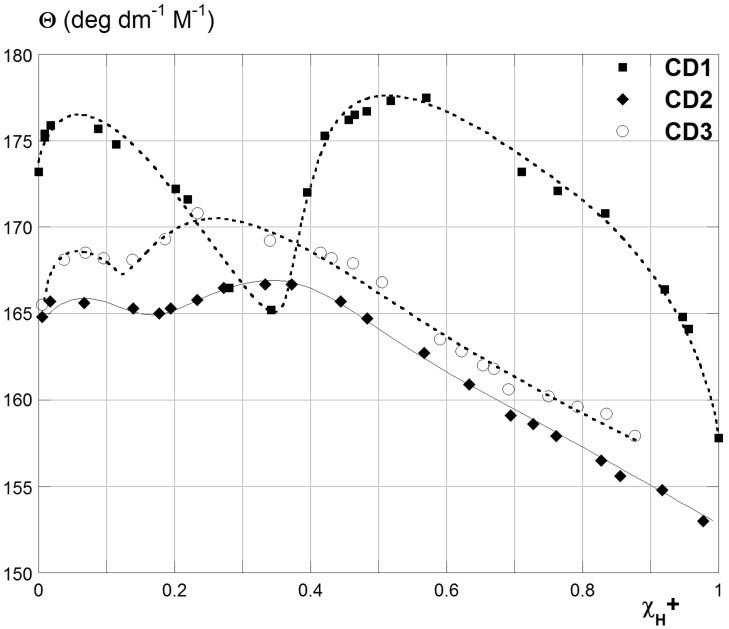
Trends of the molar optical rotation Θ of AmCDs vs χ_H+_.

Noticeably, reporting Θ vs the protonation fraction χ_H+_ (i.e., the fraction of N atoms present on average in the product which have undergone protonation at the given pH conditions, see Supporting Information File 1 for mathematical details), the absolute maxima of the three curves occur for χ_H+_ values as large as ca. 0.5, 0.33 and 0.25 for **CD1**, **CD2** and **CD3**, respectively (Figure 3). Thus, keeping into account the number of basic N atoms of the relevant polyamine branches (i.e., 2 for **A1**, 3 for **A2** and 4 for **A3**), it is immediately apparent that these maxima correspond to the situation in which on average one H^+^ has been attached to each polyamine unit. The latter observation is interesting, because it has been reported that in the case of mono-[6-(3-dimethylamino)propylamino]-6-deoxy-β-cyclodextrin the first protonation step occurs on the farthest N atom with respect to the CD cavity [48]. Thus, it is reasonable to expect that the same applies also to the polyamine branches of our AmCDs, in such a way to minimize Coulomb repulsion between cationic tail groups. Considering that the polarimetric response of CDs depends on both their intrinsic chirality and on their conformational dynamism, the behaviour observed indicates that our AmCDs experience their most extensive conformational rearrangements as the first protonation step at each polyamine branch occurs. This suggests the presence of strong intrachain interactions before protonation.

### Binding abilities of AmCDs towards *p*-nitroaniline derivatives **1**–**4**

*p*-Nitroanilines constitute a good class of probe molecules for testing the microscopic behaviour of cyclodextrins [43,49–53]. Moreover, polarimetry is a technique of choice for these systems, having been proven particularly valuable for its versatility and informative nature [51–53]. The complete polarimetric data relevant to the inclusion of guests **1**–**4** into AmCDs are collected in Supporting Information File 1 (Tables S3–S5), namely the values of the binding constants (*K*) and both the absolute (ΔΘ) and the normalized (*R*_Θ_) differential molar optical rotations as a function of the pH value and the possible presence of a buffer as supporting electrolyte. For the sake of clarity, ΔΘ is the difference between the molar optical rotations of the complex and the host, respectively, whereas *R*_Θ_ is defined as *R*_Θ_ = 100·ΔΘ/Θ (here Θ is considered at the given pH conditions). It is important to stress here that ΔΘ values for the different systems studied are not directly comparable, owing to the intrinsic differences in the absolute molar optical rotations Θ of the different hosts, and for the same host at different pH values. Thus, homogeneous comparisons can be rather carried out on the relative variations accounted for by the normalized parameter *R*_Θ_ [43]. It is also worth recalling here that ΔΘ and *R*_Θ_ values for *p*-nitroanilines are affected by an induced circular dichroism effect due to the interaction between the dipole moments of the polarized chromophore guest moiety and of the cyclodextrin cavity. Therefore, these parameters provide an estimation of the time-averaged tilt angle between the nitroaniline *C*_2_ symmetry axis and the ideal axis of the host, and serve as a good probe of the overall conformational rigidity of the complex.

**Figure 4:**
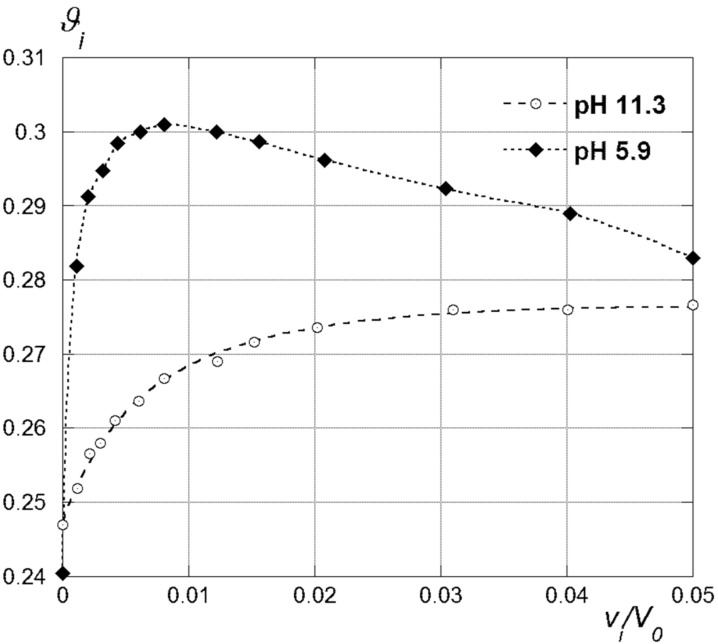
Polarimetric data trends for the inclusion of **4** in **CD1** at different pH values.

As a preliminary observation, the trends of polarimetric data (i.e., optical activities of the samples 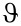
_i_ vs concentration of the guest, see Experimental) show the possible occurrence of two different behaviours (Figure 4).

With the neutral guest **1** at any pH and with the anionic guests **2**–**4** at the highest pH values, data trends account for the exclusive formation of a 1:1 host–guest inclusion complex. By contrast, with guests **2**–**4** at the lowest pH values, i.e., whenever an anionic guest meets a host bearing a significant positive charge, deviations in data trends occur on increasing the analytical concentration of the guest. These deviations indicate the formation of higher-order aggregates (see below). As long as the behaviour of host **CD1** is concerned, we observed that *K* values for the neutral guest **1** and the anionic guest **3** regularly decrease on decreasing the pH, i.e., on increasing the average charge on the host, whereas the opposite is observed with anions **2** and **4**. In all cases, however, a regular increase of R_Θ_ values generally occurs, with few exceptions. Finally, the presence of a buffer electrolyte decreases *K* values and causes significant variations of the *R*_Θ_ values. According to literature [51–53], the regular increase of *R*_Θ_ observed on decreasing the pH, indicates a concomitant decrease of the average tilt of the guest with respect to the ideal host axis. This, in turn, suggests that the polarized guest molecule penetrates more and more deeply into the CD cavity on increasing the overall positive charge, owing to the occurrence of stronger dipolar interactions. Therefore, the inclusion complex becomes stiffer and stiffer, with a consequent unfavourable effect on the complex formation entropy [43,50]. A further unfavourable contribution may also come from the increasingly difficult desolvation of the charged host. These combined effects cause the observed decrease of *K* values for the neutral guest **1**. By contrast, for the anionic guests **2** and **4** the same effects are largely counterbalanced by the concomitant occurrence of very favourable Coulomb interactions. However, the case of guest **3** is intriguing, because its trend of *K* values neatly mismatches those for the other anions. This surprising finding can be explained considering that the ancillary chain of **3** is unable to give multiple hydrogen bonding with the host cavity, due to the methyl group placed on the amino N atom. According to literature, this peculiar structural feature is able to enhance largely the outcome of entropy-unfavourable stiffening effects [50,52].

**Figure 5:**
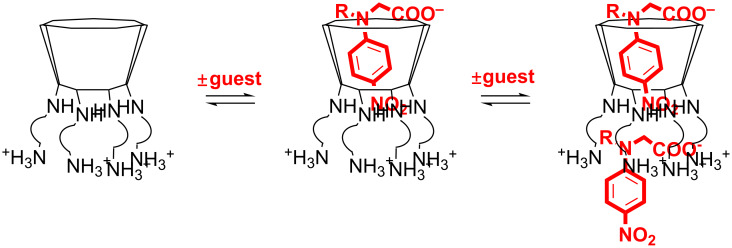
Possible association of AmCDs with guests **2**–**4**.

On the grounds of these results, the behaviour of **CD2** and **CD3** was investigated with anions **2** and **4** only. The results obtained appear quite peculiar. In fact, monoanion **2** does not present the same simple monotonic trend for *K* observed with **CD1**; by contrast, for dianion **4** the expected regular increase of *K* values on decreasing the pH is observed. These findings indicate that, on increasing the extension of the polyamine pendant bush, the outcome of favourable Coulomb effects decreases, probably due to a consequent decrease in the charge density of the bush itself. In all cases, non-monotonic *R*_Θ_ trends are found. Again, the presence of a buffering electrolyte tends to disfavour the inclusion process and significantly affects both Θ and *R*_Θ_ values, although general trends cannot be clearly envisaged. Such an effect of the electrolyte on *R*_Θ_ values is particularly interesting, because it suggests the occurrence of a significant interaction between the cationic polyamine pendant chains and the counter-anions of the buffer, probably by either ion pairing or formation of multiple hydrogen bonds. Consequently, the mobility of the chains and, in turn, the conformational dynamics of the entire CD scaffold, are significantly affected. The latter consideration may also justify the possible formation of higher order host–guest aggregates with anionic guests mentioned previously. In fact, due to molecular size, modelling considerations rule out the accommodation of more than one guest unit into the host cavity. Thus, we may reasonably hypothesize the occurrence of a loose ion-pairing external association between the cationic pendant groups of the host and the anionic guest (Figure 5).

### Binding abilities of AmCDs towards Alg

Further assessment of a possible out-of-cavity interaction for AmCDs was achieved by studying their interaction with sodium alginate (Alg). This is an easily available and stable block copolymer, constituted by β-D-mannuronate and β-L-guluronate units linked by 1→4 glycosidic bridges. Therefore, it seemed an ideal candidate as model polyanion, in order to study the stoichiometric and thermodynamic features of possible complexation with AmCDs. In particular, for our purposes the main points under examination were: i) the stoichiometry of the possible complex formed and ii) the intimate mechanism of the interaction.

**Figure 6:**
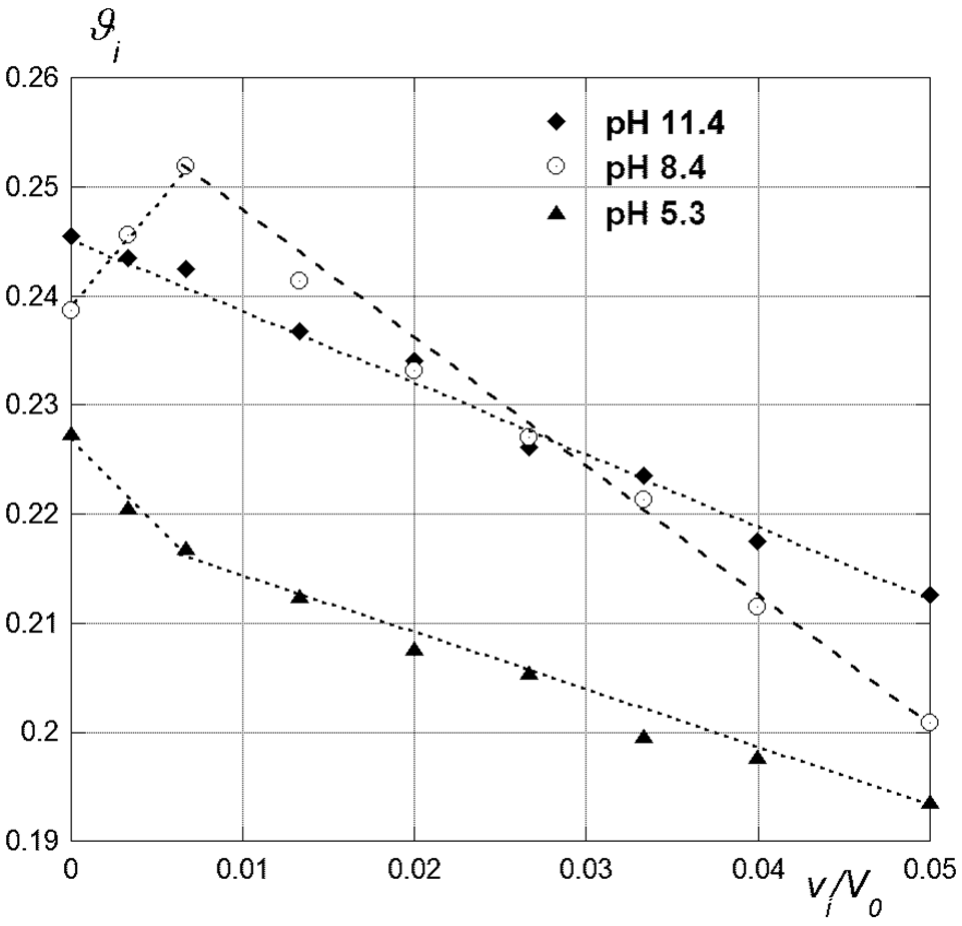
Polarimetric data trends for the **CD1**–Alg interaction (with buffer).

Series of working samples (see Experimental) were prepared at given pH values by mixing variable amounts of a concentrated solution of the polyanion (25 mN) with fixed aliquots of a solution (1.5 mM) of each AmCD. From a qualitative viewpoint, we observed three different behaviours, depending on the case. At high pH values, i.e., in the presence of the almost uncharged AmCD, the prepared samples resulted clear, irrespective of the amount of polyanion added. On reducing the pH, clear solutions were formed only at the lowest Alg concentrations, whereas an intense turbidity developed on increasing the amount of Alg beyond a limit value. On further reducing the pH down to 4.6, i.e., as the AmCD approaches its highest protonation status, the addition of Alg in any amount always caused the formation of precipitates. The latter observation suggests that in this case the added Alg is almost completely precipitated from the solution by the polycationic AmCD. This implies that a larger and larger amount of AmCD is subtracted from the solution at the same time. Noticeably, because the p*K*_a_ value reported for alginic acid is far below 4.0 [54], we can rule out that Alg is neutralized up to a significant extent under our experimental conditions. We verified that Alg alone does not form precipitates under the pH conditions used. Working samples were subjected to polarimetric analysis after centrifugation and typical trends are depicted in Figure 6.

**Figure 7:**
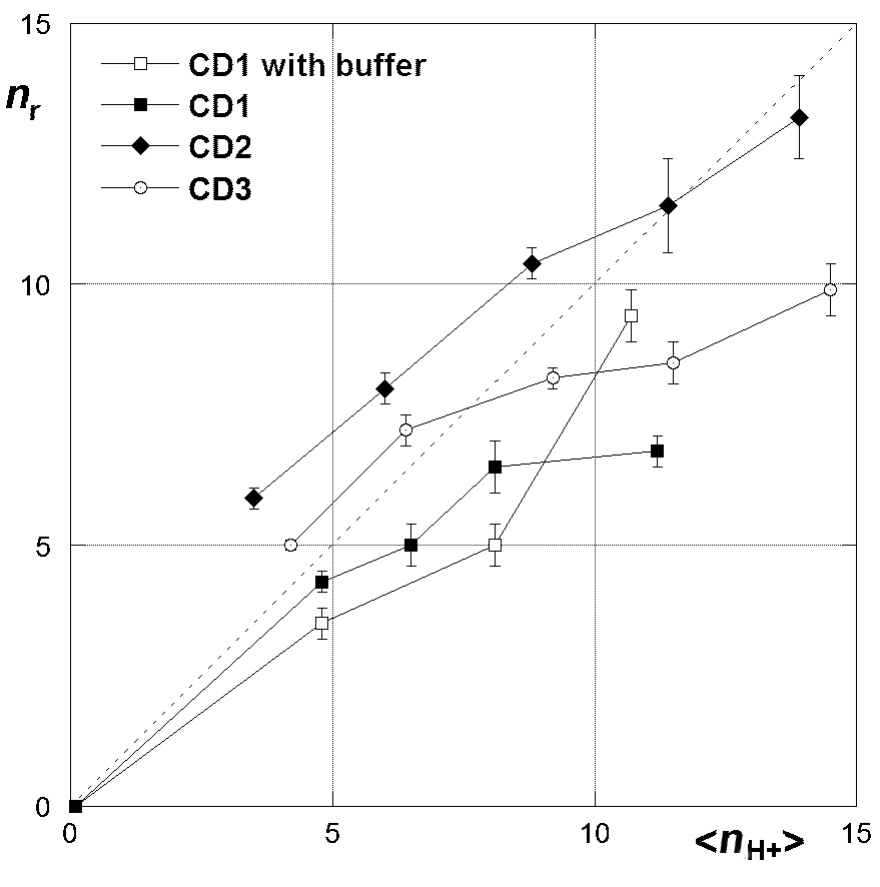
*n*_r_ Values for the AmCD–Alg interaction as a function of <*n*_H+_>.

At high pH values, we found that the optical activity observed was merely the sum of the independent contributions from AmCD and Alg. In particular, it undergoes a nearly linear decrease, owing to the fact that Alg is laevorotatory (we determined for Alg an equivalent optical activity as large as −26.3 ± 0.4 deg dm^−1^ N^−1^). This provides a convincing proof that the uncharged AmCD does not interact with the polyanion. On the other hand, for the samples prepared at lower pH values the optical activities of the supernatant liquor after centrifugation decrease accounting for the progressive subtraction of the AmCD from the solution. Indicating with *n*_r_ the average molar ratio between the AmCD and the monomer units of alginate in the precipitate formed, we derived analytically (see Supporting Information File 1 for mathematical details) the expression for the relationship between the optical activity of the samples and the amount of polyanion added (Equation 1):

[1]
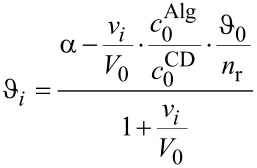


where the index i applies to the generic *i*-th sample of the series, α is a suitable intercept value, *v_i_* and *V*_0_ are the volumes of the Alg and AmCD mother solutions mixed in the sample, respectively, *c*_0_^Alg^ and *c*_0_^CD^ the relevant concentrations. Trends of the molar ratio *n*_r_ obtained as a function of the AmCD, the pH and the possible presence of a buffer electrolyte, are reported in Table 1 and depicted in Figure 7.

**Table 1:** *n*_r_ Values for the AmCD–Alg interaction.

**CD1**			**CD1** (with buffer)		

pH	<*n*_H+_>^a^	*n*_r_	pH	<*n*_H+_>^a^	*n*_r_

11.2	0.1	0	11.4*^b^*	0.1	0
8.4	4.8	4.3 ± 0.3	8.4*^c^*	4.8	3.5 ± 0.3
7.3	6.5	5.0 ± 0.2	6.5*^d^*	8.1	5.0 ± 0.4
6.5	8.1	6.5 ± 0.4	5.3*^e^*	10.7	9.4 ± 0.6
4.6	11.2	6.8 ± 0.5			

**CD2**			**CD3**		

pH	<*n*_H+_>^a^	*n*_r_	pH	<*n*_H+_>^a^	*n*_r_

10.0	3.5	5.9 ± 0.2	9.8	4.2	5.0 ± 0.1
9.0	6.0	8.0 ± 0.3	9.0	6.4	7.2 ± 0.3
7.8	8.8	10.4 ± 0.3	8.1	9.2	8.2 ± 0.2
7.0	11.4	11.5 ± 0.9	7.1	11.5	8.5 ± 0.4
6.1	13.9	13.2 ± 0.8	5.9	14.5	9.9 ± 0.5

^a^Calculated according to analytical data in Supporting Information File 1. ^b^Na_2_HPO_4_/Na_3_PO_4_ buffer (*I* = 0.1 M). ^c^B(OH)_3_/NaB(OH)_4_ buffer (*I* = 0.1 M). ^d^NaH_2_PO_4_/Na_2_HPO_4_ buffer. ^e^CH_3_COOH/CH_3_COONa buffer (*I* = 0.1 M).

It is interesting to notice that in general the *n*_r_ values never coincide with the average charge (<*n*_H+_>) on the AmCD. Thus, the precipitate must embed ions from the solution. Noticeably, **CD1** shows lower *n*_r_ values in the presence of a buffer, indicating that the amount of inorganic anions retained by the precipitate increases on increasing the content of salts in solution. On increasing their protonation status, the AmCDs appear less effective as precipitating agents on a relative scale, because *n*_r_ values are significantly larger than <*n*_H+_> at high pH values, whereas the opposite occurs at low pH. A comparison between the three different AmCDs suggests that **CD2** is the most effective ligand, showing the largest *n*_r_ values at any pH.

For the sake of completeness, we also attempted to evaluate by means of DLS measurements the size of possible AmCD–Alg aggregates. Unfortunately, very poor results were obtained. At pH 6.5, the clear supernatant liquor of the prepared samples after 24 h standing, showed the presence of no object larger than 2 nm (which corresponds, more or less, to the hydrodynamic diameter of a single AmCD unit). The same attempt made at pH 8.4 revealed the presence of a very small population of objects having an average diameter of 450 ± 150 nm. However, their concentration was so low that the relevant diffusion signal was very poor in quality. Therefore, the result found must be considered only merely indicative. However, this apparent failure is informative, because it indicates that the AmCD–Alg aggregates possess a very low ζ-potential, due to substantial charge compensation in the aggregates between the polyanion Alg, the polycationic AmCD and the buffer ions.

In order to rationalize these results, it is worth preliminarily recalling here that binding between AmCD and Alg does not involve the host cavity, but implies a different mechanism, i.e., external electrostatic and hydrogen bond interactions. Therefore, it is not comparable with the interaction with *p*-nitroanilines. Moreover, it must be considered that the three AmCDs differ for both the average number of pendant groups (<*n*_P_>) and N atoms on each CD unit. It can be reasonably hypothesized that the polyamine bush of **CD2** should experience a larger flexibility and conformational freedom as compared to **CD3** or **CD1** (see Supporting Information File 1 for further discussion of the point). Therefore, **CD2** turns out to be the best ligand towards Alg because it is able to achieve the best fitting upon the polyanion chain. We have also mentioned that protonation of the AmCD occurs first on the farthest N atoms with respect to the CD scaffold. Therefore, at relatively high pH values, charged groups on each AmCD unit benefit from a larger conformational freedom, in such a way that the ligand can interact with the polyanion in the most effective way. By contrast, at lower pH values, further positive charges on the AmCD must be allocated in the relatively narrow space around the primary cyclodextrin rim. Therefore, the increase in charge cannot much effectively improve the binding ability of the ligand, expressed in terms of number of anionic monomers per polycation unit.

### Binding abilities of AmCDs towards pUC19 and biological assays

**Figure 8:**
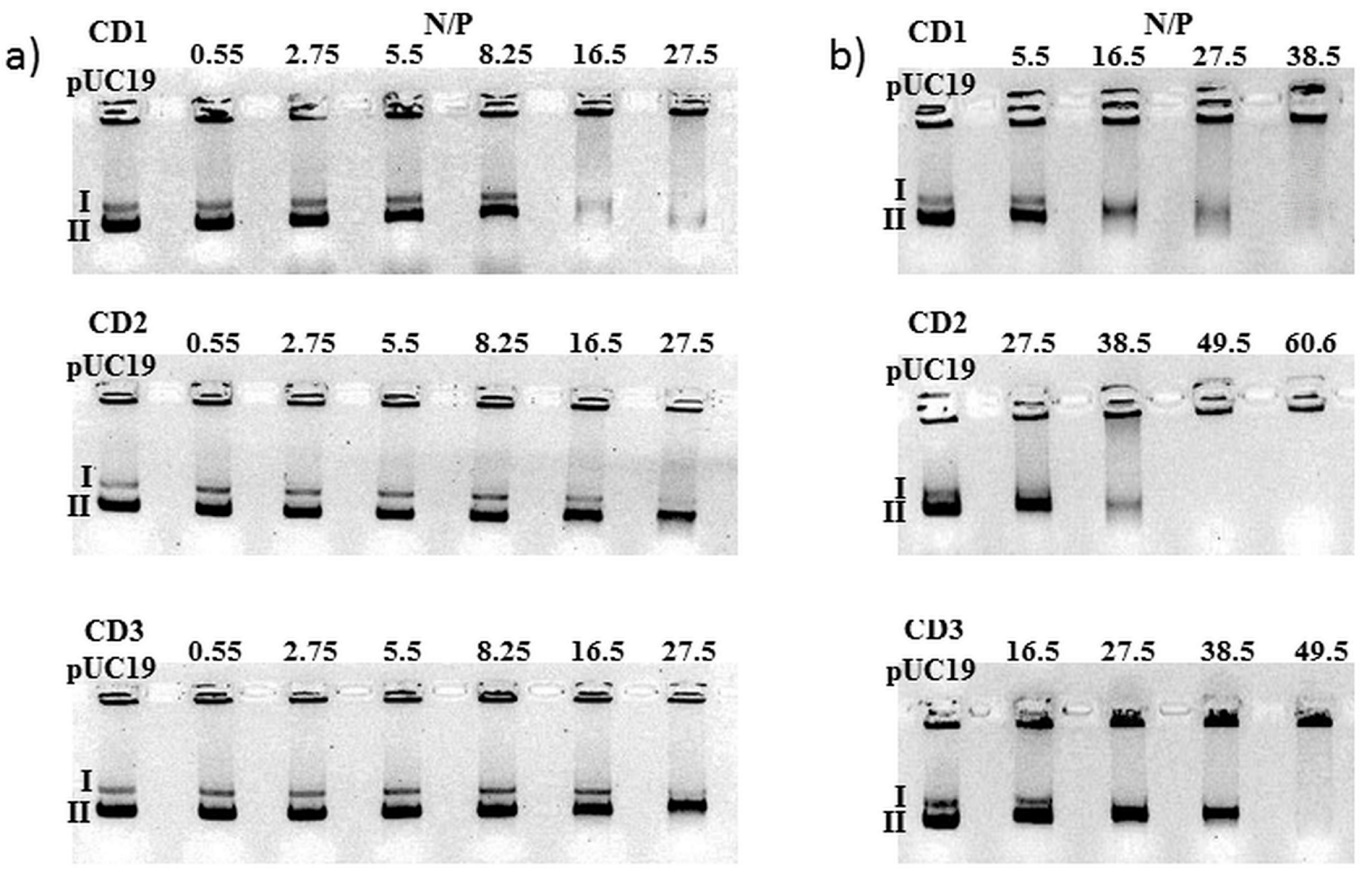
Electrophoretic mobility shift assays of pDNA in the presence of AmCDs at different N/P ratios, as indicated. **I** and **II** indicate the linear and supercoiled conformation of the pDNA, respectively. Binding is shown by the disappearance of one of the three bands: a) N/P ratios were between 0 (pUC19 only) and 27.5 for each AmCD; b) N/P ratios up to 38.5, 60.6 and 49.5 were used.

Our AmCDs also were tested for their abilities to bind plasmid DNA. For this, each AmCD was mixed with pDNA at various N/P ratios (average number of nitrogen atoms on the cyclodextrin core/number of phosphate groups of pDNA) and the complexation efficiency was evaluated by electrophoretic mobility shift assays (EMSA). When electrophoresis is applied to pDNA, different forms may be detected, i.e., the circular, linear and supercoiled topoisomers (Figure 8; in some preparations of pDNA, even after RNAse treatment, RNA can be present). Two sets of experiments were carried out: the first one (Figure 8a) with the same N/P ratios for each AmCD, and the second one with more appropriate N/P ratios (Figure 8b).

The results obtained indicate that **CD1** has the best binding abilities towards pDNA with respect to **CD2** and **CD3** (pDNA lacks migration in the gel due to AmCD binding). In fact, **CD1** bound the supercoiled conformation of pDNA almost completely at N/P 16.5 and the linear one at 38.5. Both, **CD2** and **CD3** bound the supercoiled conformation of pDNA almost completely at N/P 49.5, the linear one at 38.5 and 27.5, respectively. The lack of RNA migration occurred at N/P 16.5, 27.5 and 16.5 for **CD1**, **CD2** and **CD3**, respectively. The minimum N/P ratios for complete binding are summarized in Table 2.

**Table 2:** Minimum N/P ratios for complete binding of different forms of nucleic acids.

Nucleic acid form	**CD1**	**CD2**	**CD3**

supercoiled pDNA	16.5	49.5	49.5
linear pDNA	38.5	38.5	27.5
RNA	16.5	27.5	16.5

Differences in the apparent binding abilities of AmCDs towards pDNA may depend upon several factors affecting their mutual interaction, i.e., the state of charge, average number and length of polyamine arms of AmCDs, and the intrinsic flexibility of the polynucleotide polyanion as well. Indeed, the best performances of **CD1** with the supercoiled pDNA appear in disagreement with the results obtained with Alg. It is worth noting that, under the pH conditions used (i.e., pH 7.5), **CD1** has a lower average charge (ca. 6.2) as compared to **CD2** and **CD3** (ca. 10.4 both). Different binding efficiencies towards pDNA can be easily explained considering that, due to its peculiar conformation, the closer disposition of the negatively charged phosphate groups enables an optimization of the electrostatic interactions with the cationic AmCD. It is also reasonable to assume that the presence of the polycation may induce significant conformational changes in the fairly flexible pDNA polyanion, at the cost of inducing a certain amount of strain. Of course, these changes can hardly occur for the much stiffer Alg structure [55]. Therefore, **CD1** may overall appear more effective towards pDNA because, due to its smaller charge, binding involves a larger amount of polycation units, each inducing a relatively small strain along the polyanion chain. By contrast, the most charged **CD2** or **CD3** units cause larger strain, overall destabilizing the interaction. For the sake of completeness, the AmCD–nucleic acid complexes were subjected to the heparin challenge test in order to assess their stability. Heparin is a highly negatively charged sulfated polysaccharide, which is used as a competitor polyanion. The results obtained (see Supporting Information File 1, Figure S2) show that dissociation occurred at a heparin concentration of 400 µg/mL for **CD1** and 500 µg/mL for **CD2** and **CD3**. This indicates that the binding between DNA and AmCDs is quite strong in comparison to similar systems [28,56–57].

Finally, the effect of the three AmCDs on transformation of *E. coli* competent cells was investigated. Cyclodextrins have been already reported to change the transformation efficiency of different *E. coli* strains, although not in a standardized manner. Different cyclodextrin derivatives can cause from a 10-fold decrement up to a four-fold increment in the number of transformants compared to control experiments [58]. It is worth mentioning here that bacteria have different ways to interact with extracellular DNA. In some cases they can naturally internalize and integrate exogenous DNA (natural competence) and this can result in the acquisition of new genetic traits (e.g., antibiotic resistance genes) and the emergence of multidrug resistant strains [59]. In addition, extracellular DNA has been shown to be important for biofilm establishment and maintenance by pathogenic bacteria, such as *Pseudomonas aeruginosa* and *Staphylococcus aureus* [60–62]. Some other bacteria, such as *E. coli*, can undergo a transient period of competence after a pretreatment with calcium chloride followed by a short heat or electric shock. The addition of CaCl_2_ promotes the binding of pDNA to the outer membrane of Gram-negative bacteria. The Ca^2+^ ions both attract the negatively charged DNA backbone and neutralize the negative charges at the cell surface, thus avoiding electrostatic repulsion between the cell and the phosphate DNA groups. For other bacteria, these methods do not work well, so they hardly accept exogenous DNA and gene manipulation is hampered [63–65]. The latter cases represent a bottleneck in bacterial genetic manipulation and microbial biotechnology, since the procedures to get recombinant bacterial strains are cost and time-consuming. Thus, an increased transformation efficiency for difficult-to-manipulate strains can be very useful.

Taking into account the results obtained by EMSA, the transformation assays were performed using the following N/P ratios: 38.5 for **CD1** or 49.5 for **CD2** and **CD3**. As reference, the same amount of free pDNA was used. Surprisingly, our experiments revealed that all the AmCDs showed an adverse effect on the transformation efficiency reducing the number of bacterial colonies significantly. As a matter of fact, the transformation efficiency, considered as the number of colony forming units/µg of pDNA used (CFU), which was obtained after transformation of *E. coli* cells was as large as 1.3·10^5^ by using only the pDNA. By contrast, the CFU value dramatically decreased to approximately 5·10^2^, 2·10^2^ and 1.4·10^4^ when the complexes of pDNA with **CD1**, **CD2** and **CD3**, respectively, were used. This result suggested that the AmCDs may interact with cell membranes, e.g., by electrostatic interaction, or neutralize the anionic nature of pDNA, making the addition of calcium chloride in the preparation of competent cells pointless.

## Conclusion

The binding abilities of AmCDs as bimodal supramolecular ligands were successfully tested towards neutral and anionic *p*-nitroaniline derivatives, sodium alginate and the pUC19 plasmid DNA, chosen as suitable models. First, regarding the inclusion of small-sized guests into the CD scaffold cavity, we verified that the process is largely affected by the protonation status of the host, as a function of the pH, and by the possible presence of buffering electrolytes. The main factors ruling the binding efficiency are Coulomb interactions, medium effects and conformational restraints, with a subtle interplay determining the occurrence of non-linear data trends in some cases. Therefore, the behavior observed and the relevant rationalization proposed by us, appear perfectly consistent with the previous literature on the topic [50]. Second, our materials are able to form stable aggregates with polyanions such as alginate or pDNA. In these cases, of course, binding implies an outer-sphere interaction of the polyanion with the polyamine cationic “bush”, so it cannot be compared with the interaction with *p*-nitroanilines. As long as Alg is concerned, analysis of the composition of the aggregates, as accounted for by *n*_r_ values, enabled us to evidence i) the effect of inorganic electrolytes, which ensure charge counterbalance, resulting in low ζ-potential and precipitation and ii) the effect of the structure of the polyamine branches, in term of both their pH-dependent overall charge and their conformational freedom. Third, the interactions of AmCDs with pDNA, studied by means of EMSA and transformation of *E. coli* Ca-competent cells assays, revealed a very strong binding, which ultimately hampers the desired cellular uptake.

As a further remark, we can outline that the latter apparently negative result is quite intriguing indeed, because we may envisage a possible use of our materials as scavengers of extracellular DNA (eDNA). As a matter of fact, recent studies have shown that eDNA is important for biofilm establishment and maintenance by pathogenic bacteria, such as *P. aeruginosa* and *S. aureus* [60–62]. In addition, eDNA with its negative charge can sequester cationic antibiotics contributing to antibiotic resistance; thus, removing eDNA from the biofilm matrix can weaken the biofilm and can raise its susceptibility to antibiotics. The fact that these cyclodextrin derivatives might be loaded with an antibiotic allows speculating that a possible antibiotic–CD complex could target the pathogen and in the meanwhile to bind and sequester extracellular DNA, inhibiting its role in vivo. Finally, a particular mention is deserved to the fact that, once again, simple polarimetry proves to be a versatile and powerful tool for the study of supramolecular interactions, even in situations where quite complex systems are involved.

## Experimental

### Materials

All the reagents needed were used as purchased (Sigma, Aldrich), without further purification. The sodium alginate sample used (Sigma, lot 077K1583, extracted from *Macrocystis pyrifera*), presented an average content of mannuronic and guluronic units of 61% and 39%, respectively, and a molecular weight in the range 70–100 kDa. The AmCDs **CD1**–**CD3** [37] were prepared by solvent-free aminolysis at 60 °C for 48 h of the heptakis(6-deoxy-6-iodo)-β-CD with a 140-fold mole-to-mole excess of the proper linear polyamine, i.e., 3-(*N*,*N*-dimethylamino)propylamine (**A1**) for **CD1**, bis(3-aminopropyl)methylamine (**A2**) for **CD2** and bis-1,2-[(3-aminopropyl)amino]ethane (**A3**) for **CD3**. The products were isolated and purified by repeated precipitations from ethanol/diethyl ether. The characterization was carried out by means of potentiometric acid–base titration, which confirmed that the ionization behaviour of the AmCDs can be modelled as a mixture of four independent virtual weak bases. The results obtained were in full agreement with the values reported in the cited reference (relevant discussion and mathematical details are reported in Supporting Information File 1). The *p*-nitroanilines **1**–**3** [50] were prepared by nucleophilic aromatic displacement reaction between 4-nitrofluorobenzene and a slight excess (ca. 10%) of the proper amine in DMSO at 70 °C for 4 hours in the presence of Na_2_CO_3_ (1 equiv). The same procedure was adapted for the synthesis of guest **4**.

#### *N*-(4-Nitrophenyl)iminodiacetic acid disodium salt (**4**)

Iminodiacetic acid (1.33 g, 10 mmol) was treated with an equimolar amount of sodium methoxide, obtained by dissolving metallic sodium (0.46 g, 20 mmol) in dry methanol (20 mL), and the mixture was distilled in vacuo (Rotavapor). The residue was dissolved in dry DMSO (10 mL), then, 4-nitrofluorobenzene (1.41 g, 10 mmol) and anhydrous Na_2_CO_3_ (1.06 g, 10 mmol) were added. The reaction mixture was kept at 70 °C under stirring for 18 hours. Afterwards, the resulting slurry was dissolved in water (200 mL) and the solution was extracted twice with ethyl acetate (ca. 70 mL each). Then, the aqueous phase was acidified with HCl (6 M) up to pH 2, and the desired product was extracted thrice with ethyl acetate (100 mL each). The organic extracts were dried over Na_2_SO_4_ and distilled in vacuo (Rotavapor) to afford the crude product, which was dissolved in a methanolic solution of sodium methoxide (2 M, 10 mL). Diethyl ether (80 mL) was then added to precipitate the pure product, which was finally filtered off. Yield 60% (1.79 g). IR (nujol) ν (cm^−1^): 1597, 1516, 1339; ^1^H NMR (300 MHz, D_2_O) δ (ppm) 3.95 (s, 4H, -CH_2_-), 6.47 and 8.03 (2d, *J* = 9.5 Hz, 2H + 2H, *p*-NO_2_-C_6_H_4_-N<); Anal. calcd for C_10_H_8_N_2_O_6_Na_2_: C, 40.28; H, 2.70; N, 9.40; Na, 15.42; found: C, 40.24; H, 2.73; N, 9.39; Na, 15.43.

#### Polarimetry

Polarimetric determinations were performed on a JASCO P-1010 polarimeter. In order to obtain molar optical rotations Θ of AmCDs at different pH values, 1.5 mM mother solutions of the materials in double-distilled water were prepared. Then a small volume of either standard HCl (1 M) or NaOH (1 M) was added to aliquots (3 mL each) of the solutions, in order to adjust the pH to the desired value. From the observed optical rotations of the samples (corrected for the small dilution effect), the relevant Θ values were easily calculated. Measurements of the binding constants for guests **1**–**4** were accomplished according to the general procedure described elsewhere [51,53]. In brief, 1.5 mM stock solutions of the hosts were prepared by dissolving the proper amount of substance either in pure water followed by adjusting the pH at the desired value by adding small amounts of HCl (1 M) or NaOH (1 M), or in an aqueous buffer solution at the desired pH value (the buffers used are specified in the footnote of Tables S3–S5, in Supporting Information File 1). Then, sets of sample solutions were prepared by mixing variable amounts (up to 150 μL) of a concentrated guest solution (usually ca. 0.25 M) to fixed volumes (3 mL) of the host solution. In each case, the actual pH value of the solutions was checked with a pH-meter. Polarimetric data were subjected to regression analysis by means of the proper equation derived analytically [51,53]. Good fitting provides convincing evidence that only 1:1 host–guest complexes are present in the samples. Otherwise, deviations from the expected trend give evidence of the formation of higher order aggregates.

In order to study the interaction between AmCDs and Alg, a stock alginate solution 25 mN was first prepared as follows. The proper amount of substance (99.5 mg) was dissolved in warm water and after cooling to rt the volume was adjusted to 20 mL. Finally, the solution was filtered through a 0.45 μ Millipore^®^ filter. For the sake of clarity, the concentration (normality) was calculated according to the formula weight of the monomer unit (C_6_H_7_O_6_Na). Then, sets of samples were prepared by mixing fixed aliquots (3 mL each) of stock AmCD solutions at the proper pH value with increasing micro-amounts of Alg solution. Each sample was vigorously shaken, allowed to settle overnight and then centrifuged at 5000 rpm for 15 min. The supernatant liquors were carefully pipetted and the relevant optical activities were measured. Data were finally subjected to regression analysis according to Equation 1 (the relevant mathematical details are reported in Supporting Information File 1).

#### Dynamic light scattering

DLS measurements to evaluate the possible dimensions of AmCD–Alg aggregates were performed with a Malvern Instruments Zetasizer NANO-ZS apparatus. The intensity of the diffused light was evaluated at 173°; autocorrelation functions were analysed by means of the cumulants method using the Sasfit 0.94.4 software package. Samples were prepared in aqueous buffer at either pH 6.5 or pH 8.4, by mixing the proper amounts of Alg with each AmCD in such a way to have concentrations as large as 0.25 mN for Alg and 1.5 mM for the AmCD. The turbid mixtures obtained were allowed to settle for 24 h and then, the apparently clear supernatant liquors were carefully pipetted and analysed.

#### Biological assays

*E. coli* strain DH10B and the plasmid pUC19 (pDNA) were used in this study. *E. coli* was grown at 37 °C in Luria broth (LB) or terrific broth (TB) liquid medium. When necessary, solid medium was obtained by adding agar to the liquid medium. Standard genetic techniques with *E. coli* and in vitro DNA manipulations were performed as described previously [66–67]. The pUC19 (ca. 2700 bp, conferring ampicillin resistance to *E. coli*) was extracted by alkaline lysis method [64]. Electrophoretic mobility shift assays (EMSA) of the pDNA were performed as described elsewhere [68]. For EMSA experiments, constant amounts (200 ng) of pDNA were incubated for 30 minutes at room temperature in a final volume of 20 µL of binding buffer 1× (12.5 mM Tris HCl pH 7.5, 10% glycerol, 62.5 mM KCl, 0.75 mM DTT), using different N/P ratios (average number of nitrogen atoms on the cyclodextrin core/number of phosphate groups of pDNA). The pH value of the reaction medium was kept at 7.5 using TE buffer as the solvent [59]. Ten µL of each binding reaction sample (mixed with bromophenol blue dye at a 1:1 ratio) was loaded on a 0.8% agarose gel containing ethidium bromide in TBE 0.5× (5.4 g Tris Base, 2.75 g boric acid, 2 mL of EDTA, 0.5 M, pH 8) pH 7.5 along with the pUC19 DNA as reference. At the end of the electrophoresis, the gels were visualized under UV light using a Bio-Rad Trans illuminator. The illuminated gels were photographed by using a Polaroid camera.

Heparin competitive displacement assays were carried out as follows: AmCD–pDNA complexes at selected N/P ratios (N/P = 40 for **CD1**, N/P = 50 for **CD2** and **CD3**) were first prepared as described earlier, and were subsequently added with increasing concentrations of heparin sodium salt solution from porcine intestinal mucosa. The mixtures were incubated at room temperature for 30 min. Afterwards, the solution was analysed by 0.8% agarose gel electrophoresis to examine the released pDNA from complexes.

Calcium competent *E. coli* cells were prepared as follows: *E. coli* cells were grown in LB medium at 37 °C up to an OD_650_ of 0.6. The cells were collected by centrifugation, resuspended in a 70 mM CaCl_2_ solution and incubated on ice for 1 hour. Then, the cells were collected by centrifugation, treated with freezing solution (70 mM CaCl_2_/10% glycerol (w/v)), aliquoted and finally stored at −80 °C. Transformation of calcium competent *E. coli* cells was performed by heat shock treatment using 20 µL of different mixtures AmCD/pDNA at selected N/P ratios (N/P = 40 for **CD1**, N/P = 50 for **CD2** and **CD3**), as well as with free pDNA. The cells were incubated on ice for 30 min, for 45 min at 42 °C and again on ice for 2 min. Then, 500 µL of LB medium were added to each mixture and cells were incubated at 37 °C for 1 hour. Aliquots of 50 µL of each transformation were put on a Petri disc containing LB agar medium supplemented with 100 µg/mL of ampicillin to select *E. coli* cells containing the plasmid. Bacterial growth was allowed overnight at 37 °C. The experiment was performed in triplicate. The number of vital bacteria was considered as the colony forming units (CFU) obtained in presence of AmCD–pDNA complexes or free pDNA.

## Supporting Information

Supporting information features a mechanistic scheme for the synthesis of AmCDs and discussion of their synthesis and characterization; analytical and polarimetric data for AmCDs; mathematical details about polarimetric determination of binding constants and about Equation 1 and heparin challenge tests.

File 1 Further experimental information.
