# Supplementary material for: Binding abilities of polyaminocyclodextrins: polarimetric investigations and biological assays
Source: Beilstein J Org Chem. 2017 Dec 18;13:2751–63. doi: 10.3762/bjoc.13.271 (PMC5753052; doi:10.3762/bjoc.13.271)
Supplement: File 1 — Further experimental information. [file Beilstein_J_Org_Chem-13-2751-s001.pdf]

Supporting Information

for

Binding abilities of polyaminocyclodextrins: polarimetric investigations and biological assays

Marco Russo[1], Daniele La Corte[1], Annalisa Pisciotta[1], Serena Riela[1], Rosa Alduina[1], and Paolo Lo Meo*[1,2]

Address: [1]Dipartimento di Scienze e Tecnologie Biologiche, Chimiche e Farmaceutiche (STEBICEF), University of Palermo, V.le delle Scienze ed. 17, 90128 Palermo, Italy and [2]ATeNCenter, University of Palermo, V.le delle Scienze ed. 18, 90128 Palermo, Italy

Email: Paolo Lo Meo - paolo.lomeo@unipa.it

* Corresponding author

Further experimental information

Table of Contents

- **Figure S1** Mechanistic scheme for polysubstitution processes in the synthesis of AmCDs.
- Notes on the characterization of AmCDs and their structures.
- **Table S1** Analytical data for AmCDs.
- **Table S2** Molar optical rotations Θ of materials **CD1**–**CD3** as a function of the pH.
- **Table S3** Polarimetric data for the inclusion of guests **1**–**4** in **CD1**.
- **Table S4** Polarimetric data for the inclusion of guests **2** and **4** in **CD2**.
- **Table S5** Polarimetric data for the inclusion of guests **2** and **4** in **CD3**.
- Notes on the derivation of equation 1.
- **Figure S2** Heparine challenge tests.

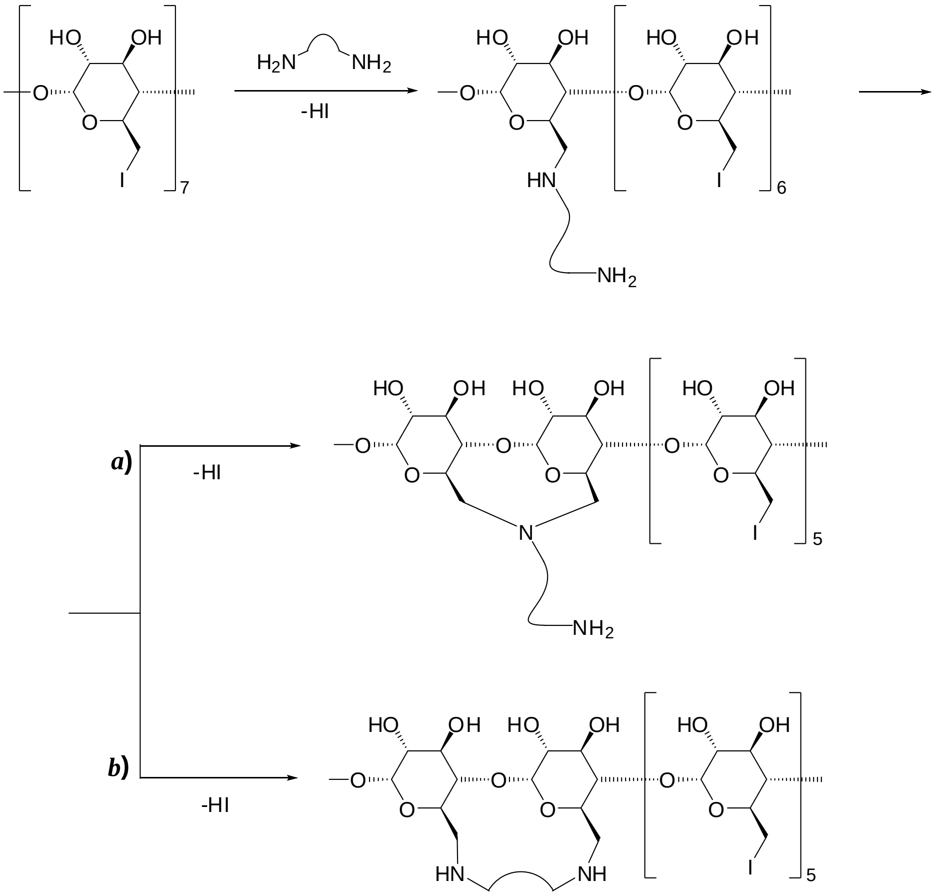

Figure S1. Mechanistic scheme for polysubstitution processes in the synthesis of **AmCDs**

Notes on the characterization of AmCDs and their structures

As mentioned in the main paper, the potentiometric characterization of materials **CD1**–**CD3** was carried out as described in the literature [1] In brief, a weighed amount of the material was dissolved in double-distilled water and placed in the presence of an excess HCl; then the solution was titrated with conc. NaOH, and the titration curve obtained was subjected to regression analysis by means of the proper equation derived analytically, i.e.:

$$\frac{v_i}{V_o} = \frac{\frac{n_{HCl}+n_{HI}}{V_o} + \frac{K_w}{10^{-pH}} - 10^{-pH} - \sum_{i=1}^{4}\left(\frac{n_{B(i)}}{V_o}\cdot\frac{10^{-pH}}{10^{-pH}+K_{B(i)H^+}}\right)}{c_{NaOH}+10^{-pH}-\frac{K_w}{10^{-pH}}}$$

This equation has been obtained by modelling the behaviour of the AmCDs as a mixture of four independent fictitious weak bases. For the sake of clarity, this means that the titration curve obtained for the materials is indistinguishable (within the limit of experimental

uncertainties) from the one that would be obtained from a mixture of four weak bases. The relevant analytical data obtained are collected in Table S1:

Table S1: Analytical data for **AmCD**s from potentiometric titration.

		CD1	CD2	CD3
**B(1)**	$\chi_{\mathbf{B(1)}}$	0.23 ± 0.02	0.31 ± 0.02	0.24 ± 0.02
	$pK_{\mathbf{B(1)}H^+}$	5.8 ± 0.3	5.8 ± 0.1	5.1 ± 0.1
**B(2)**	$\chi_{\mathbf{B(2)}}$	0.26 ± 0.02	0.25 ± 0.01	0.24 ± 0.01
	$pK_{\mathbf{B(2)}H^+}$	6.8 ± 0.1	7.4 ± 0.1	6.9 ± 0.1
**B(3)**	$\chi_{\mathbf{B(3)}}$	0.33 ± 0.02	0.19 ± 0.01	0.28 ± 0.02
	$pK_{\mathbf{B(3)}H^+}$	8.8 ± 0.1	8.9 ± 0.1	8.8 ± 0.1
**B(4)**	$\chi_{\mathbf{B(4)}}$	0.18 ± 0.02	0.25 ± 0.01	0.24 ± 0.03
	$pK_{\mathbf{B(4)}H^+}$	10.2 ± 0.1	10.4 ± 0.1	10.4 ± 0.1
	$<\boldsymbol{n_p}>$	5.7 ± 0.3	6.1 ± 0.1	4.5 ± 0.2
	$<\boldsymbol{n_{HI}}>$	4.0 ± 0.5	5.1 ± 0.2	6.0 ± 0.5

It is important to stress that the four weak bases have no real physical meaning. Of course, the overall equivalent of basic nitrogen atoms in the weighed sample equals the sum of the equivalents of the fictitious bases. Hence, the average number of N atoms and polyamine pendants per AmCD unit can be calculated by trivial algebraic passages.

From the regression analysis of the titration curves, the apparent molar fractions ($\chi_{B(i)}$) and dissociation constants ($K_{B(i)H+}$) of the virtual bases are obtained. From these data, it is possible to calculate at any pH value the protonation fraction, the average number of H^+ bound, and therefore the average positive charge per AmCD unit, by means of the following expressions, which can be obtained by simple algebraic passages:

$$\chi_{H+} = \sum_{i}\left(\chi_{B(i)}\frac{10^{-pH}}{10^{-pH}+K_{B(i)H^+}}\right) \quad \text{and} \quad <\boldsymbol{n_H}\mathbf{+}> = <\boldsymbol{n_p}>\cdot \mathbf{n}_N\cdot\chi_{H+}$$

where n_N is the number of nitrogen atoms of the polyamine chains.

A further comment is deserved to the results relevant to the average number of polyamine pendants per AmCD unit, i.e., 5.7, 6.1 and 4.5 for **CD1**, **CD2** and **CD3**, respectively. Indeed, the latter finding implies that the probability of multiple substitution for the three different polyamines increases in the order **A2** < **A1** < **A3**. The fact that the lowest average number of pendants is found for the polyamine **A3**, having the largest number of N atoms, suggests that in this case multiple substitution mainly occurs through different N atoms of the same polyamine chain, according to path “b” shown in Figure S1. Consequently, the conformational freedom of the polyamine pendants of **CD3** must experience significant

restrictions. By contrast, for **A2** multiple substitution seems to occur preferentially on the same N atom, according to path “a” in Figure S1. Therefore, the polyamine pendants of **CD2** should benefit of the largest conformational freedom. For **A1** path “*a*” is of course the only possibility.

As a final remark, we reported elsewhere [1-3] that it is possible to evidence for each AmCD product, by means of high-resolution ESIMS techniques, the presence of the various components of the mixture bearing a different number of polyamine branches per cyclodextrin unit. In particular, the *m*/*z* values obtained provide convincing proof of the molecular formulas relevant to the various possible derivatives. Noticeably, in ESIMS spectra the possible presence of cyclodextrin dimers is never detected. Therefore, the formation of dimers or oligomers in appreciable amounts under the synthetic conditions used can be reasonably ruled out. Attempts to separate adequately the different components of the mixtures by means of HPLC techniques were unsuccessful.

For **CD1** we found the following signals (*m*/*z*): 862.5605 $[C_{77}H_{154}N_{14}O_{28}{\cdot}2H]^{2+}$ (calcd 862.5601);. 811.5020 $[C_{72}H_{140}N_{12}O_{28}{\cdot}2H]^{2+}$ (calcd 811.5023).

For **CD2** we found the following signals (*m*/*z*): 1013.2068 $[C_{91}H_{189}N_{21}O_{28}{\cdot}2H]^{2+}$ (calcd 1013.2078);. 940.6287 $[C_{84}H_{170}N_{18}O_{28}{\cdot}2H]^{2+}$ (calcd 940.6289); 868.0492 $[C_{77}H_{151}N_{15}O_{28}{\cdot}2H]^{2+}$ (calcd 868.0495).

For **CD3** we found the following signals (*m*/*z*): 1114.8004 $[C_{98}H_{210}N_{28}O_{28}{\cdot}2H]^{2+}$ (calcd 1114.8007); 1027.7085 $[C_{90}H_{188}N_{24}O_{28}{\cdot}2H]^{2+}$ (calcd 1027.7085); 940.6166 $[C_{82}H_{166}N_{20}O_{28}{\cdot}2H]^{2+}$ (calcd 940.6163); 853.5242 $[C_{74}H_{144}N_{16}O_{28}{\cdot}2H]^{2+}$ (calcd 853.5241).

Table S2: Molar optical rotations Θ of **CD1–CD3** as a function of the pH.

**CD1**			**CD2**			**CD3**		
pH	$\langle n_{H^+} \rangle$[a]	Θ[b] (deg dm^{-1} M^{-1})	pH	$\langle n_{H^+} \rangle$[a]	Θ[b] (deg dm^{-1} M^{-1})	pH	$\langle n_{H^+} \rangle$[a]	Θ[b] (deg dm^{-1} M^{-1})
12.2	0.0	173.2	12.2	0.1	164.8	12.0	0.1	165.5
11.6	0.1	175.2	11.5	0.3	165.7	11.2	0.7	168.1
11.5	0.1	175.4	10.9	1.2	165.6	10.8	1.3	168.5
11.1	0.2	175.9	10.3	2.5	165.3	10.6	1.8	168.2
10.4	1.0	175.7	10.1	3.2	165.0	10.3	2.6	168.1
10.1	1.3	174.8	10.0	3.5	165.3	10.0	3.5	169.3
9.5	2.3	172.2	9.7	4.2	165.8	9.7	4.4	170.8
9.4	2.5	171.6	9.4	4.9	166.5	9.0	6.4	169.2
9.1	3.2	166.5	9.0	6.0	166.7	8.6	7.8	168.5
8.8	3.9	165.2	8.7	6.7	166.7	8.5	8.1	168.2
8.6	4.5	172.0	8.2	8.0	165.7	8.3	8.7	167.9
8.4	4.8	175.3	7.9	8.7	164.7	8.0	9.5	166.8
8.2	5.2	176.2	7.4	10.2	162.7	7.3	11.1	163.5
8.1	5.3	176.5	7.0	11.4	160.9	7.0	11.7	162.8
8.0	5.5	176.7	6.6	12.5	159.1	6.8	12.3	162.0
7.7	5.9	177.3	6.4	13.1	158.6	6.7	12.6	161.8
7.3	6.5	177.8	6.2	13.7	157.9	6.5	13.0	160.6
6.5	8.1	173.2	5.9	14.9	156.5	6.1	14.1	160.2
6.3	8.7	172.1	5.7	15.4	155.6	5.7	14.9	159.6
5.9	9.5	170.8	5.3	16.5	154.8	5.4	15.7	159.2
5.4	10.5	166.4	4.7	17.6	153.0	5.1	16.5	157.9
5.2	10.8	164.8						
5.1	10.9	164.1						
3.8	11.4	157.8						

[a]Calculated according to analytical data of Table S1. [b]All data are given with a ± 0.5% indetermination.

Table S3: Polarimetric data for the inclusion of guests **1–4** in **CD1**.

guest	buffer [a]	pH	$\langle n_{H+} \rangle$ [b]	K (M^{-1})	$\Delta\Theta$ [c] (deg dm^{-1} M^{-1})	R_Θ
**1**	-	5.1	10.9	280 ± 40[d]	46.4 ± 2.6	28.3 ± 1.6
	-	6.3	8.7	340 ± 70[d]	47.3 ± 3.3	27.5 ± 1.9
	-	8.0	5.5	1000 ± 100[d]	37.6 ± 0.9	21.3 ± 0.5
	-	9.5	2.3	1070 ± 130[d]	35.3 ± 3.5	20.5 ± 2.0
	-	11.6	0.1	1220 ± 140[d]	36.6 ± 0.7	20.9 ± 0.4
	**P1**	6.5	8.1	(<50)	n.d.	n.d.
	**B**	8.4	4.8	400 ± 20	52.2 ± 1.2	30.1 ± 0.7
	**Am**	9.2	2.9	580 ± 50	45.1 ± 1.0	26.3 ± 0.6
	**P2**	11.3	0.2	1200 ± 140	38.8 ± 1.0	23.1 ± 0.6
**2**	-	5.4	10.5	1490 ± 160[d]	50.6 ± 1.2	30.4 ± 0.7
	-	6.5	8.1	1180 ± 250[d]	54.5 ± 2.0	31.5 ± 1.2
	-	8.1	5.3	1020 ± 190[d]	57.2 ± 1.6	32.4 ± 0.9
	-	9.5	2.3	960 ± 130[d]	44.3 ± 1.0	25.7 ± 0.6
	-	11.5	0.1	490 ± 50[d]	43.9 ± 1.0	25.0 ± 0.6
**3**	-	5.1	10.9	(<50)	n.d.	n.d.
	-	7.7	5.9	520 ± 80	88.6 ± 4.2	50.0 ± 2.4
	-	8.4	4.8	780 ± 160	64.4 ± 3.1	37.2 ± 1.8
	-	10.1	1.3	1030 ± 30	47.2 ± 0.3	27.0 ± 0.2
	-	11.5	0.1	1220 ± 140	36.6 ± 0.7	20.9 ± 0.4
**4**	-	6.3	8.7	(> 10^4)	n.d.	n.d.
	-	8.4	4.8	1300 ± 480	23.9 ± 1.4	13.8 ± 0.8
	-	9.1	3.2	520 ± 70	22.8 ± 0.5	13.7 ± 0.3
	-	11.1	0.2	67 ± 15	29.9 ± 2.6	17.0 ± 1.5
	**P1**	5.9	9.5	(> 10^4)	(51 ± 10)	(31 ± 6)
	**B**	8.4	4.8	350 ± 30	21.7 ± 0.4	13.0 ± 0.2
	**Am**	9.2	2.9	300 ± 40	22.0 ± 0.6	13.4 ± 0.4
	**P2**	11.3	0.2	190 ± 20	24.1 ± 0.6	14.6 ± 0.4

[a]Buffers used (I = 0.1 M) are specified as follows: **Ac**, CH_3COOH/CH_3COONa; **Am**, NH_4Cl/NH_3; **B**, $B(OH)_3/NaB(OH)_4$; **P1**, NaH_2PO_4/Na_2HPO_4; **P2**, Na_2HPO_4/Na_3PO_4; “-” indicates no buffer. [b]Calculated according to analytical data reported in **Table S1**. [c]Data given within a ± 0.5 deg dm^{-1} M^{-1} indetermination. [d]Data from ref. [1], reported for useful comparison.

Table S4: Polarimetric data for the inclusion of guests **2** and **4** in **CD2**.

guest	buffer[a]	pH	$\langle n_{H^+} \rangle$ [b]	K (M^{-1})	$\Delta\Theta$ [c] (deg dm^{-1} M^{-1})	R_{Θ}
**2**	-	5.7	15.4	1070 ± 100	51.0 ± 0.9	32.7 ± 0.6
	-	7.0	11.4	860 ± 40	54.9 ± 0.6	34.1 ± 0.4
	-	8.7	6.7	790 ± 70	49.0 ± 1.0	29.4 ± 0.6
	-	10.1	3.2	520 ± 40	50.8 ± 1.0	30.8 ± 0.6
	**Ac**	4.7	17.6	430 ± 20	44.1 ± 0.7	29.4 ± 0.5
	**Ac**	5.7	15.4	730 ± 100	45.4 ± 1.4	27.5 ± 0.8
	**P1**	6.3	13.4	530 ± 70	50.9 ± 1.4	30.8 ± 0.8
	**B**	8.6	7.0	490 ± 60	53.4 ± 1.7	31.4 ± 1.0
	**P2**	10.8	1.3	540 ± 30	53.4 ± 0.6	31.2 ± 0.4
**4**	-	5.7	15.4	(> 10^4)	n.d.	n.d.
	-	6.6	12.5	4300 ± 600	32.1 ± 0.6	20.2 ± 0.4
	-	7.9	8.7	1350 ± 140	26.0 ± 0.3	15.8 ± 0.2
	-	8.7	6.7	1310 ± 90	25.5 ± 0.5	15.3 ± 0.3
	-	10.0	3.5	610 ± 90	32.2 ± 1.3	19.5 ± 0.8
	**Ac**	5.6	15.8	(> 10^4)	(37 ± 8)	(22 ± 5)
	**P1**	6.2	13.7	4800 ± 500	32.9 ± 1.9	19.2 ± 1.1
	**B**	8.5	7.2	320 ± 20	25.6 ± 0.5	14.7 ± 0.3
	**P2**	10.9	1.2	200 ± 30	37.0 ± 1.0	21.3 ± 1.0

[a]Buffers used (I = 0.1 M) are specified as follows: **Ac**, CH_3COOH/CH_3COONa; **Am**, NH_4Cl/NH_3; **B**, $B(OH)_3/NaB(OH)_4$; **P1**, NaH_2PO_4/Na_2HPO_4; **P2**, Na_2HPO_4/Na_3PO_4; "-" indicates no buffer. [b]Calculated according to analytical data reported in **Table S1**. [c]Data given within a ± 0.5 deg dm^{-1} M^{-1} indetermination.

Table S5: Polarimetric data for the inclusion of guests **2** and **4** in **CD3**.

**2**	-	5.4	15.7	980 ± 30	52.0 ± 0.3	32.7 ± 0.2
	-	6.8	12.3	1110 ± 60	56.2 ± 0.7	34.6 ± 0.4
	-	8.5	8.1	1500 ± 200	48.4 ± 1.1	28.8 ± 0.7
	-	10.6	1.8	290 ± 30	57.9 ± 2.3	34.4 ± 1.4
	**P1**	5.1	16.5	450 ± 30	43.5 ± 0.7	27.1 ± 0.4
	**P1**	6.7	12.6	1580 ± 170	49.2 ± 1.0	28.5 ± 0.6
	**B**	8.3	8.7	750 ± 90	47.3 ± 1.3	26.6 ± 0.7
	**P2**	10.3	2.6	450 ± 30	48.1 ± 1.7	27.9 ± 0.4
**4**	-	6.1	14.1	(> 10^4)	(40 ± 8)	(25 ± 5)
	-	7.0	11.7	2700 ± 200	35.6 ± 0.4	21.9 ± 0.3
	-	8.6	7.8	1170 ± 40	28.2 ± 0.1	16.7 ± 0.1
	-	10.6	1.8	360 ± 30	31.4 ± 0.7	18.7 ± 0.4
	**P1**	5.7	14.9	6000 ± 2000	31.4 ± 1.6	18.9 ± 1.0
	**P1**	6.7	12.6	2000 ± 200	23.5 ± 1.7	13.6 ± 1.0
	**B**	8.3	8.7	640 ± 50	23.8 ± 0.4	13.4 ± 0.2
	**P2**	10.8	1.3	73 ± 7	35.5 ± 1.0	20.6 ± 0.6

[a]Buffers used (I = 0.1 M) are specified as follows: **Ac**, CH_3COOH/CH_3COONa; **Am**, NH_4Cl/NH_3; **B**, $B(OH)_3/NaB(OH)_4$; **P1**, NaH_2PO_4/Na_2HPO_4; **P2**, Na_2HPO_4/Na_3PO_4; "-" indicates no buffer. [b]Calculated according to analytical data reported in **Table S1**. [c]Data given within a ± 0.5 deg dm^{-1} M^{-1} indetermination.

Notes on the derivation of equation 1

Considering first the cases at low pH values, i.e., when a precipitate is always formed in all the working samples, it is immediately evident that the optical rotation of the generic *i*-th sample ϑ_i will be due to the residual AmCD present in solution. This will be given by the difference between the initial amount of AmCD (n_0^{CD}) and the amount of AmCD co-precipitated with the alginate ($n_{(cp)}$). In turn, the co-precipitated AmCD will be bound to the amount of alginate added ($n_{(\mathbf{Alg})}$), according to the relationship: $n_{(cp)} = n_{(\mathbf{Alg})}/n_r$. On the other hand, it must be:

$n_0^{CD} = c_0^{CD} \cdot V_0$ and $n_{(Alg)} = c_0^{Alg} \cdot v_i$

Then:

$$\vartheta_i = \Theta[\text{AmCD}]_i = \Theta \cdot \frac{n_0^{CD} - n_{(cp)}}{v_i + V_0} = \Theta \cdot \frac{n_0^{CD} - n_{(Alg)}/n_r}{v_i + V_0} =$$

$$= \Theta \cdot \frac{c_0^{CD} V_0 - \dfrac{c_0^{Alg} \cdot v_i}{n_r}}{v_i + V_0} = \Theta \cdot \frac{c_0^{CD} - \dfrac{c_0^{Alg}}{n_r} \cdot \dfrac{v_i}{V_0}}{1 + \dfrac{v_i}{V_0}}$$

On the other hand, the optical rotation ϑ_0 for the solution without **Alg** must be: $\vartheta_0 = \Theta\, c_0^{CD}$.
Then: $\Theta = \vartheta_0/c_0^{CD}$, which inserted in the previous expression gives:

$$\vartheta_i = \frac{\vartheta_0 - \dfrac{c_0^{Alg}}{n_r} \cdot \dfrac{v_i}{V_0}}{1 + \dfrac{v_i}{V_0}}$$

The latter expression is a particular form of equation 1. It is intuitively evident that in the samples prepared at intermediate pH values, after a saturation level is reached and precipitate formation takes place, the functional relationship between ϑ_i and v_i/V_0 must be formally the same as the one provided by the last expression. The only difference must lie in the fact that in the latter case the intercept of the function coincides with ϑ_0 because precipitation starts immediately. By contrast, when precipitation does not start immediately, the "regular" trend matter-of-factly undergoes a translation, which can be simply accounted for by introducing an intercept α different from ϑ_0. This substitution leads to equation 1.

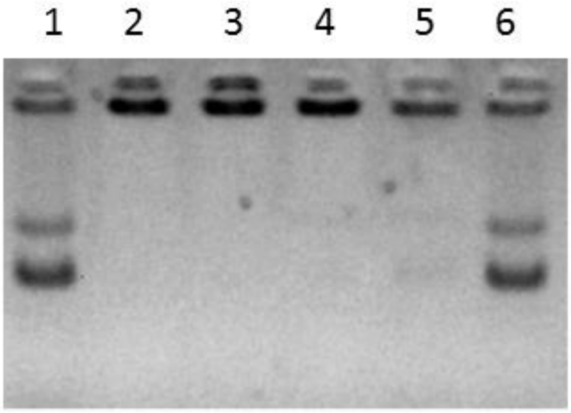

1. pUC19
2. pUC19+ CD1
3. pUC19+ CD1+100 µg/ml Hep
4. pUC19+ CD1+200 µg/ml Hep
5. pUC19+ CD1+300 µg/ml Hep
6. pUC19+ CD1+400 µg/ml Hep

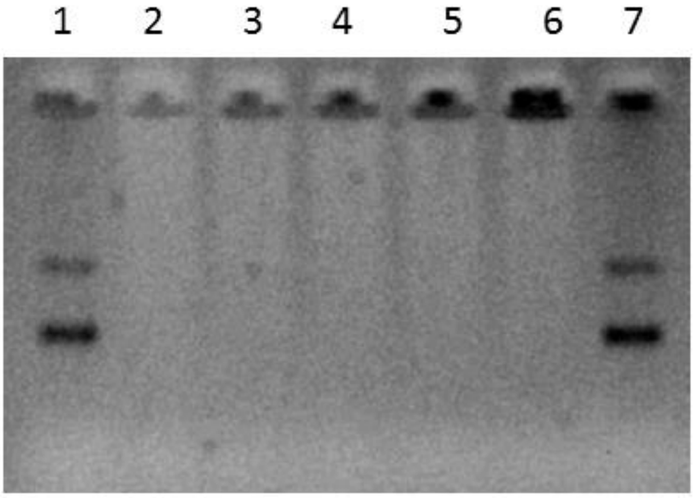

1. pUC19
2. pUC19+ CD2
3. pUC19+ CD2+100 µg/ml Hep
4. pUC19+ CD2+200 µg/ml Hep
5. pUC19+ CD2+300 µg/ml Hep
6. pUC19+ CD2+400 µg/ml Hep
7. pUC19+ CD2+500 µg/ml Hep

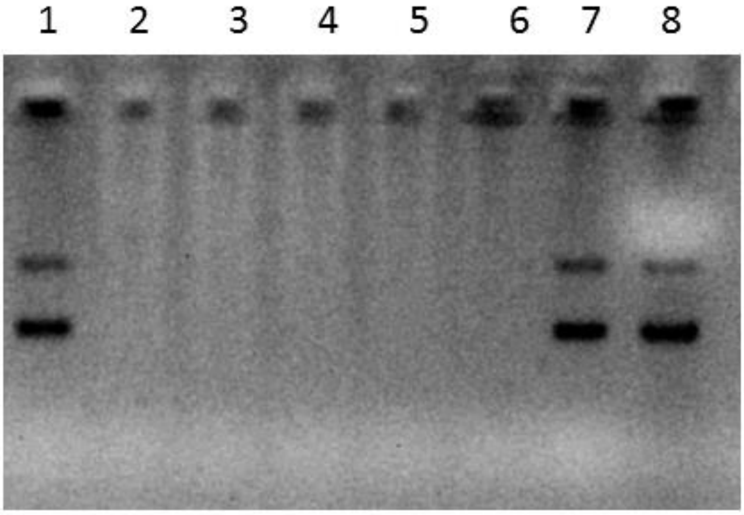

1. pUC19
2. pUC19+ CD3
3. pUC19+ CD3+100 µg/ml Hep
4. pUC19+ CD3+200 µg/ml Hep
5. pUC19+ CD3+300 µg/ml Hep
6. pUC19+ CD3+400 µg/ml Hep
7. pUC19+ CD3+500 µg/ml Hep
8. pUC19+ CD3+600 µg/ml Hep

Figure S2: Heparine challenge tests.

References

1. Lo Meo, P.; D'Anna, F.; Gruttadauria, M.; Riela, S.; Noto, R. *Carbohydr. Res.* **2012**, *347*, 32.
2. Russo, M.; Meli, A.; Sutera, A.; Gallo, G.; Chillura Martino, D.; Lo Meo, P.; Noto, R. *RSC Adv.* **2016**, *6*, 40090.
3. Russo, M.; Saladino, M. L.; Chillura Martino, D.; Lo Meo, P.; Noto, R. *RSC Adv.* **2016**, *6*, 49941.
